# Analysis of turnover intention and influencing factors among female nurses with two children in Grade A tertiary public hospitals in Sichuan province: a cross-sectional study

**DOI:** 10.3389/fpubh.2024.1416215

**Published:** 2024-08-22

**Authors:** Chengrong Ling, Liande Tao, Xin Wang, Yunlian Wu, Yong Chai, Lan Zhang

**Affiliations:** ^1^Department of Nursing, Jinzhou Medical University, Jinzhou, Liaoning, China; ^2^Department of Nursing, The Second People’s Hospital of Yibin, Yibin, Sichuan, China; ^3^Department of Nursing, Huaian Hospital of Huaian City, Huaian, Jiangsu, China; ^4^Department of Nursing, First Affiliated Hospital of Jinzhou Medical University, Jinzhou, Liaoning, China

**Keywords:** female nurses, second children, work–family conflict, regulatory emotional self-efficacy, turnover intention

## Abstract

**Objective:**

This study aims to examine the current status of turnover intention among female nurses with two children and explore the factors influencing their decision to resign, ultimately providing a basis for reducing nurses’ turnover intention and stabilizing the nursing workforce.

**Methods:**

A convenience sampling method was used to select 1,370 in-service female nurses with two children from 65 Grade A tertiary public hospitals in Sichuan Province from September to December 2023. Data was collected through a general information questionnaire, work-family behavioral role conflict scale, regulatory emotional self-efficacy, and turnover intention scale.

**Results:**

This study revealed that the average score for turnover intention among female nurses with two children was (13.11 ± 3.93). There was a positive correlation between work-family behavioral role conflict and turnover intention (*r* = 0.485, *p* < 0.01), while regulatory emotional self-efficacy showed a negative correlation with turnover intention (*r* = −0.382, *p* < 0.01). The main influencing factors for resignation among these nurses included age, number of night shifts per month, average monthly income, primary caregiver for children, work-to-family conflict and family-to-work conflict, and the ability to express positive emotions (POS), the capacity to regulate negative emotions such as despondency/distress (DES), and the skill to manage anger/irritation (ANG). Collectively, these factors explained 29.5% of the total variance in turnover intention scores.

**Conclusion:**

Turnover intention among female nurses with two children is relatively high. To address this issue, hospital managers shall implement effective measures through various channels to settle work–family conflict, enhance nurses’ regulatory emotional self-efficacy, and reduce turnover intention resulting from work–family conflict. Together, these efforts will reduce nurse turnover and foster a stable nursing workforce.

## 1 Introduction

In recent years, public hospitals have experienced unprecedented transformations due to technological advancements and societal progress. However, the global shortage of nurses and the high turnover in the nursing industry are increasingly severe (1). In fact, the turnover intention among nursing personnel in China’s tertiary public hospitals has reached a staggering 22.02% (2), which poses significant challenges to the thriving development of public hospitals. Turnover intention refers to the likelihood of individuals switching jobs within a specific timeframe and serves as a psychological state and a precursor to actual resignation, which provides valuable insights into future resignation behavior (3).

With the increasing number of families with two children, nurses in the role of “mothers of two children” have to bear the pressure of housework and childcare after raising a second child, as well as the special occupational pressure they have to endure in the current state of heavy nursing workload and relative shortage of personnel (4, 5). As a result, two-child female nurses may be more likely to leave their jobs out of consideration for their responsibilities to their families, their children, and their heavy nursing workloads. Paying attention to the willingness of female nurses with two children to leave their jobs and the factors affecting them, and preventing the wastage of female nurses with two children are of great significance in stabilizing the team of nurses in public hospitals and carrying out nursing work in an orderly manner. Turnover intention can be influenced by numerous factors, with research indicating that work–family conflict is a crucial contributing factor (6). Work–family conflict is a specific type of role conflict, which is based on role conflict theory (7). It refers to individuals who have temporarily uncoordinated roles in two very different domains, work and family, at the same time, and need to give up one of the roles in order to fulfil the other (8). In the long run, this will result in an imbalance of the role pressure and the formation of work–family conflict. It is characterized by specific events of behavioral conflicts, where work encroaches upon family life or family obligations interfere with work commitments. The presence and intensity of work-family behavioral role conflict reflect the degree of work–family conflict experienced by individuals (9).

Due to the high intensity and frequent night shifts associated with nursing work, it is more susceptible to work–family conflict and turnover intention, especially among nurses with children. Female nurses who have given birth to two children at different points in time, at the same time take care of the child and go out to work to earn economic income (10). In the current state of heavy nursing workload and relative shortage of personnel, female nurses who have had two children are experiencing higher levels of work–family conflict due to increased responsibility for family child care, and the conflict and overlap of time between the work and family spheres consumes more of the nurses’ energy (5, 11), resulting in higher levels of work–family conflict among female nurses who have had two children (12). Specifically, female nurses who have given birth to and are raising two children face the challenge of balancing childcare responsibilities with the need to generate income through employment (13). As reported by international media (14), the turnover intention of married social workers is more subject to work–family conflict. Domestic research (15) in China has also found a positive correlation between the level of work–family conflict and turnover intention. As the healthcare service demands of the population continue to evolve, the development of nursing in China faces significant opportunities and challenges. Under such high pressure, nurses often experience negative occupational emotions such as low job satisfaction, reduced wellbeing, and occupational burnout (16).

Gender theory typically delves into the differences in roles, expectations, behaviors, and opportunities between men and women in society. As society evolves, the concept of gender equality has gradually gained widespread acceptance. However, in the workplace, particularly in the medical field, gender roles and societal expectations can still impact the turnover intentions of nurses, especially those who are second-time mothers (17). In traditional concepts, women are often expected to bear more family responsibilities, including taking care of children and household chores. Such societal expectations can create pressure on second-time mothers who are also nurses, as they need to cope with both heavy work and the demands of caring for multiple children. When this pressure reaches a certain level, it may lead to their intention to quit their jobs in order to better balance their family and work life.

Bandura proposed the concept of emotion regulation self-efficacy in 2003, which is the degree to which an individual is confident that he or she can effectively regulate his or her own emotional state, and is a sense of the individual’s ability to manage his or her own emotional state (18). Research has shown (19) that individuals with high regulatory emotional self-efficacy are more likely to experience job satisfaction, thereby reducing their turnover intention. This study is based on the psychological stress theory proposed by Jiang (20). The theory suggests that life events can affect the mind and body by either directly causing stress or indirectly acting on the individual through mediating factors. The birth of a second child distracts nurses from their limited time and energy, adding to the conflicts and contradictions between roles in both the work and family spheres. Role conflict can lead to stress and negative emotions in individuals, and emotion regulation self-efficacy can help individuals to better cope with these negative effects, thus reducing the willingness to leave. In this study, the work-family role conflict brought about by the second child was used as a serious life event stressor (21), and emotion regulation self-efficacy, as a coping resource, can help individuals better cope with the stress and reduce the willingness to leave the job, which is a result of the psychological stress response (22).

Currently, most studies of nurses’ willingness to leave have focused on populations such as emergency department nurses (23) and male nurses (24), and few studies have focused on the willingness to leave of the special group of two-child female nurses. In China, the researches on nurses’ willingness to leave started late, and most of the studies on nurses’ willingness to leave in China used self-designed questionnaires, and the results obtained lacked scientific validity. And researches on influencing factors has been limited by focusing on the field of demographics. Most of the studies on willingness to leave involve psychological and social aspects, and the level of nurses’ willingness to leave is not exactly the same in different hospital grades in different geographic regions, and the factors affecting willingness to leave are also different. Female nurses with two children, who typically possess a certain amount of work experience and qualifications, constitute the core workforce responsible for ensuring clinical nursing quality and safety. They are the driving force behind the development of the nursing workforce, and it is crucial for every hospital manager to prioritize their physical and mental wellbeing and retain them in the workplace.

Therefore, this study aimed to understand the current situation of the willingness to leave among two-child female nurses in a tertiary-level public hospital in Sichuan Province, and to analyse the effects of work–family conflict and emotion regulation self-efficacy on the willingness to leave. It provides a theoretical basis and direction for early detection of their willingness to leave and the development of targeted career planning measures to further reduce the willingness of two-child female nurses to leave in tertiary kilometer hospitals and to stabilize the nursing workforce in public hospitals. Managers can take appropriate measures to rationalize the deployment of human resources and provide psychological interventions and positive stress reduction to reduce nurses’ intention to leave, develop a stable nursing workforce, and promote the high-quality development of public hospitals.

## 2 Subjects and methods

### 2.1 Subjects

A convenience sampling method was employed to select 1,392 female nurses who had given birth to a second child and met the inclusion criteria from 65 Grade A tertiary public hospitals in 21 cities and prefectures in Sichuan Province, China from September to December 2023. The inclusion criteria were as follows: (1) Female registered nurses working in hospitals, (2) more than 1 year in clinical nursing, (3) two children, both alive, (4) informed consent and voluntary participation in research. Nurses with the following conditions were excluded from the study: (1) Regulatory nurses, (2) nurses for multiple pregnancies, (3) nurses whose marital status is divorced or widowed, (4) nurses in non-clinical nursing positions or nurses who have been out of clinical nursing for more than 6 months.

### 2.2 Research tools

#### 2.2.1 General information questionnaire

The general information questionnaire was designed by the researchers based on the study objectives and a review of the literature. It included items such as the participant’s hospital, age, professional title, educational degree, department, position, employment status, spouse’s occupation, years of work experience, monthly income, number of night shifts per month, age and gender of the first child, age and gender of the second child, whether the second child was a planned pregnancy, primary caregiver for the children, and total family income.

#### 2.2.2 Work-family behavioral role conflict scale

The scale in this study was developed by Clark (9). It is used to rate the level of role conflict between work and family behavior. Translated into Chinese by Sun et al. (25) and applied to a population of Chinese nurses (26). It consists of two dimensions: work-to-family conflict and family-to-work conflict. The scale consists of 30 items, each of which is rated on a 5-point Likert scale ranging from 1 to 5 on a scale from “completely disagree” to “completely agree,” with a total score of 30–150. Higher total scores indicate higher levels of work–family conflict, while lower scores show lower levels of work–family conflict. The Cronbach’s α coefficient for this scale in the current study was determined to be 0.952.

#### 2.2.3 Regulation emotional self-efficacy scale

The regulatory emotional self-efficacy scale used in this study was originally developed by Caprara et al. (27). It was primarily used to assess nurses’ emotional regulation self-efficacy. Translated and revised for localization by Wen et al. (28) and widely used in the Chinese nurse population (29–31). The scale measures three dimensions: the ability to express POS, the capacity to regulate negative emotions such as DES, and the skill to manage ANG. It comprises a total of 12 items, with responses ranging from “completely inconsistent” to “completely consistent,” scored on a scale of 1–5, respectively. Higher scores indicate a stronger sense of regulatory emotional self-efficacy, while lower scores suggest lower levels of regulatory emotional self-efficacy for the subjects. The Cronbach’s α coefficient for this scale in the current study was tested to be 0.967.

#### 2.2.4 Turnover intention scale

The turnover intention scale was originally developed by Michaels and Spedtor (32) in 1982 to measure individuals’ levels of intention to resign. Translated into Chinese by Li and Li (33) and widely used in the Chinese nurse population (34–36). The scale consists of three dimensions and six items: turnover intention I, turnover intention II, and turnover intention III, with each dimension comprising two items. Each item is scored on a scale of 1–4, with higher scores implying stronger intentions to resign. Specifically, scores of “≤1” show extremely low intentions, scores between “>1 and ≤ 2” note relatively low intentions, scores between “>2 and ≤ 3” signify relatively high intentions, and scores above “3” indicate very high intentions to resign. In this study, the Cronbach’s *α* coefficient was examined to be 0.838.

### 2.3 Data collection process and ethical approval

The study was approved by the Ethics Committee of the Second People’s Hospital of Yibin City (Approval number: 2023-225-01), and the ethical guidelines stipulated by the Ethics Committee were strictly followed.

For this study, a web-based questionnaire was selected as the data collection method. The research tool was integrated into the Wenjuanxing platform to generate QR codes and links for distribution. The researchers established contact with various hospitals, typically reaching out to individuals such as deputy directors, directors of nursing departments, and head nurses. They explained the study’s purpose, methods, and significance and received support from these officials. Subsequently, a training session was conducted for a designated staff member (nursing department officer) at each participating hospital regarding instructions on questionnaire distribution and survey administration. The training content encompassed the survey’s objectives, guidelines for completing the questionnaire, and important notes during the process. Prior to conducting the research study, nurses who met the inclusion criteria were provided with a statement explaining in detail the purpose, content and significance of the study. This statement is easy to understand, and after obtaining the participant’s consent, the participant signs an informed consent form informing and committing the nurse that the information obtained from this survey is limited to this study and will not be used for any other purpose. Participation in the survey was voluntary, confidential, and anonymous, with participants being encouraged to seek clarification from the researchers if needed. The data will be destroyed after this study.

There are 26 variables in the questionnaire in this study. According to the rough sample estimation method proposed by Kendall, the sample size can be 5–10 times of the variables. A sample size of 156–312 cases was derived, taking into account a 20% sample attrition and convenience sampling error. To ensure more comprehensive and valuable data, a larger sample size was used, with multiple centres included in the study, resulting in a final sample of 1,392 participants. After excluding 22 invalid questionnaires, a total of 1,392 questionnaires were collected. The final dataset consisted of 1,370 valid questionnaires with a response rate of 98.42%.

### 2.4 Statistical methods

SPSS 27.0 statistical software was used for data analysis. A significance level of *p* < 0.05 was set to determine statistical significance. Categorical data was described by frequency and percentage while scale scores were reported by mean ± standard deviation. Pearson correlation analysis was conducted to explore the relationship between work-family behavioral role conflict, regulatory emotional self-efficacy and turnover intentions. Independent sample *t*-tests and one-way ANOVA were performed to compare the differences in turnover intention scores among female nurses with two children of different demographic characteristics. Multiple linear regression analysis was employed to identify the factors influencing turnover intentions.

## 3 Results

### 3.1 Turnover intentions, work-family behavioral role conflict, and regulatory emotional self-efficacy among female nurses with two children

Among female nurses with two children, the mean item score for work-family behavioral role conflict was (2.77 ± 0.64). The average item score for work–family conflict was (3.38 ± 0.72). The average item score for family–work conflict was (2.16 ± 0.74). Overall, the level of work-family behavioral role conflict was moderately low (35). The mean item score for regulating emotional self-efficacy was (3.68 ± 0.65). The average item scores for the different dimensions were as follows: POS, 4.16 ± 0.71; DES, 3.51 ± 0.79, and ANG, 3.37 ± 0.85. Collectively, the level of regulatory emotional self-efficacy was moderately high (37). In addition, the average item score of turnover intention was (2.18 ± 0.66). The average item scores for the different dimensions of turnover intention were as follows: turnover intention I (1.94 ± 0.86), turnover intention II (2.33 ± 0.71), and turnover intention III (2.28 ± 0.65). Turnover intentions were relatively high (38). Please refer to Table 1 for more detailed information.

**Table 1:** Scores for work-family behavioral role conflict, regulatory emotional self-efficacy, and turnover intention (*n* = 1,370).

Project	Number of items (items)	Theoretical maximum score	Score (score, mean ± SD)	Average item score (Score, mean ± SD)
Work-family behavioral role conflict	30	150	83.01 ± 19.29	2.77 ± 0.64
Work-to-family conflict	15	75	50.63 ± 10.81	3.38 ± 0.72
Family-to-work conflict	15	75	32.38 ± 11.17	2.16 ± 0.74
Regulatory emotional self-efficacy	12	60	44.18 ± 7.79	3.68 ± 0.65
POD dimension	4	20	16.63 ± 2.83	4.16 ± 0.71
DES dimension	4	20	14.05 ± 3.15	3.51 ± 0.79
ANG Dimension	4	20	13.5 ± 3.42	3.37 ± 0.85
Turnover intention	6	24	13.11 ± 3.93	2.18 ± 0.66
Turnover intention I dimension	2	8	3.88 ± 1.71	1.94 ± 0.86
Turnover intention II dimension	2	8	4.67 ± 1.41	2.33 ± 0.71
Turnover intention III dimension	2	8	4.56 ± 1.29	2.28 ± 0.65

### 3.2 One-way ANOVA on turnover intentions among female nurses with two children

To examine the differences in turnover intentions among female nurses with two children of different demographic characteristics, independent sample *t*-tests and one-way ANOVA were conducted. The results, presented in Table 2, revealed statistically significant differences in turnover intention scores among nurses with varying ages, professional titles, years of work experience, night shifts per month, average monthly income, age of their first child, age of their second child, and primary caregiver for their children (*p* < 0.05). Only the items that exhibited statistically significant differences are reported in this study.

**Table 2:** Comparison of turnover intention among female nurses with two children of different demographic characteristics (*n* = 1,370).

Project	Group	Score (score, mean ± SD)	*F-*value	*P*	LSD
Age	<30^a^	14.06 ± 4.01	7.176^F^	<0.001	a > b/c/d/e
	31 ~ 35^b^	13.44 ± 3.88			b > c/d/e
	36 ~ 40^c^	12.92 ± 4.06			c > d/e
	41 ~ 45^d^	12.17 ± 3.41			
	>45^e^	10.59 ± 3.29			
Professional title	Junior and below^a^	13.42 ± 4.14	4.444^F^	0.012	a/b > c
	Intermediate^b^	13.09 ± 3.86			
	Senior and above^c^	12.11 ± 3.55			
Years of work experience	Years<5^a^	13.00 ± 3.68	5.044^F^	<0.001	b > c/d/e
	5 ≤ years<10^b^	13.90 ± 3.8			c > e
	10 ≤ years<15^c^	13.25 ± 3.91			
	15 ≤ years<20^d^	12.79 ± 4.02			
	≥20^e^	12.11 ± 3.81			
Night shifts per month	No nights shifts^a^	12.39 ± 3.87	10.860^F^	<0.001	a < b/c/d
	1 ~ 5^b^	13.36 ± 3.93			b < d
	6 ~ 10^c^	13.39 ± 3.86			c < d
	>10^d^	14.79 ± 3.87			
Average monthly income (Yuan)	<3,500^a^	14.88 ± 4.03	5.951^F^	<0.001	a > b/c/d
	3,501 ~ 5,500^b^	13.55 ± 4.19			b > c/d
	5,501 ~ 10,000^c^	12.92 ± 3.83			
	>10,000^d^	12.5 ± 3.54			
Age of the first child	<3^a^	14.69 ± 4.42	9.359^F^	<0.001	a > d
	3 ~ <6^b^	14.18 ± 3.78			b > c/d
	6 ~ <10^c^	13.25 ± 3.92			c > d
	≥10^d^	12.59 ± 3.89			
Age of the second child	<3^a^	13.48 ± 3.93	5.577^F^	0.001	a > c/d
	3 ~ <6^b^	13.26 ± 3.91			b > c/d
	6 ~ <10^c^	12.7 ± 3.93			c > d
	≥10^d^	11.29 ± 3.64			
Primary caregiver for the children	Oneself^a^	13.24 ± 4.31	2.570^F^	0.025	b < e
	Oneself and spouse^b^	12.78 ± 3.74			d < e
	Hired nanny^c^	12.78 ± 4.1			
	Paternal grandparents^d^	12.9 ± 3.81			
	Maternal grandparents^e^	13.65 ± 4.09			
	Others^f^	14.08 ± 3.96			

The options for each group are assigned to sort a–f.

### 3.3 Correlation analysis of work-family behavioral role conflict, regulatory emotional self-efficacy, and turnover intentions among nurses with two children

The study discovered a positive correlation between work-family behavioral role conflict and turnover intentions among nurses with two children (*r* = 0.485, *p* < 0.01). Additionally, a negative correlation was observed between regulatory emotional self-efficacy and turnover intentions (*r* = −0.382, *p* < 0.01). Please refer to Table 3 for detailed information.

**Table 3:** Correlation analysis of work-family behavioral role conflict, regulatory emotional self-efficacy, and turnover intentions among nurses with two children (*n* = 1,370, *r-*value).

Variables	Work-to-family conflict	Family-to-work conflict	WFBRCS score	POS	DES	ANG	RESE score	Total score of RIS
Work-to-family conflict	1	–	–	–	–	–	–	–
Family-to-work conflict	0.540^**^	1	–	–	–	–	–	–
Work-family behavioral role conflict	0.873^**^	0.882^**^	1	–	–	–	–	–
POS	−0.148^**^	−0.195^**^	−0.196^**^	1	–	–	–	–
DES	−0.346^**^	−0.294^**^	−0.364^**^	0.249^**^	1	–	–	–
ANG	−0.343^**^	−0.294^**^	−0.362^**^	0.263^**^	0.482^**^	1	–	–
Regulatory emotional self-efficacy	−0.381^**^	−0.352^**^	−0.418^**^	0.641^**^	0.82^**^	0.807^**^	1	–
Turnover intention	0.412^**^	0.440^**^	0.485^**^	−0.239^**^	−0.311^**^	−0.301^**^	−0.382^**^	1

^*^P < 0.01,^**^P < 0.01. POS, express positive affect; DES, regulate manage despondency/distress affects; ANG, regulate manage anger/irritation affects.

### 3.4 Multivariate linear regression analysis of factors influencing turnover intentions among nurses with two children

To further investigate the factors influencing turnover intentions among female nurses with two children, a multivariate linear regression analysis was performed. The analysis included variables that showed statistically significant differences in the one-way ANOVA, as well as the dimensions of the WFBRCS and the RESE, as independent variables. The total score of turnover intentions was used as the dependent variable. The significant variables identified in the one-way ANOVA were age, professional title, years of work experience, night shifts per month, average monthly income, age of the first child, age of the second child, and primary caregiver for the children. For categorical variables, dummy variables and numerical variables were set to directly incorporate into the multivariate linear regression analysis. Detailed information regarding variable assignments can be found in Table 4.

**Table 4:** Independent variables and variable assignments.

Independent variables	Variable assignments
Age	<30 years old = 1;31 ~ 35 = 2;36 ~ 40 = 3;41 ~ 45 = 4;>45 = 5
Professional title	Junior and below (X1 = 0, X2 = 0, X3 = 0); Intermediate (X1 = 0, X2 = 1, X3 = 0); Senior and above (X1 = 0, X2 = 0, X3 = 1)
Years of work experience	<5 Years = 1;5 ≤ Years<10 = 2;10 ≤ Years<15 = 3;15 ≤ Years<20 = 4;≥20 Years =5
Night shifts per month	0 = 1;1 ~ 5 = 2;6 ~ 10 = 3;>10 = 4
Average monthly income	<3, 500 Yuan = 1;3, 501 ~ 5, 500 Yuan =2;5, 501 ~ 10, 000 Yuan = 3;>10, 000 Yuan = 4
Age of the first child	<3 years old = 1;3 ~ <6 = 2;6 ~ <10 = 3;≥10 = 4
Age of the second child	<3 = 1;3 ~ <6 = 2;6 ~ <10 = 3;≥10 = 4
Primary caregiver for the children	Oneself (X1 = 0, X2 = 0, X3 = 0, X4 = 0, X5 = 0, X6 = 0); Oneself and spouse (X1 = 0, X2 = 1, X3 = 0, X4 = 0, X5 = 0, X6 = 0); Hired nanny (X1 = 0, X2 = 0, X3 = 1, X4 = 0, X5 = 0, X6 = 0); Paternal grandparents (X1 = 0, X2 = 0, X3 = 0, X4 = 1, X5 = 0, X6 = 0); Maternal grandparents (X1 = 0, X2 = 0, X3 = 0, X4 = 0, X5 = 1, X6 = 0); Others (X1 = 0, X2 = 0, X3 = 0, X4 = 0, X5 = 0, X6 = 1)
All dimensions in work-family behavioral role conflict scale and regulation emotional self-efficacy scale	Plugging in the original values

The results of a multiple linear regression analysis revealed that nine variables included the regression equation to explain the turnover intention score. These variables were age, night shifts per month, average monthly income, primary caregivers being the child’s grandparents, family–work conflict, work–family conflict, POS, DES, and ANG. The regression model yielded an *R*^2^ value of 0.299. After adjustment, *R*^2^ = 0.295, *F* = 64.579, and *p* < 0.001. These variables accounted for 29.5% of the total variation in the score of turnover intention, as shown in Table 5.

**Table 5:** Multivariate linear regression analysis of factors influencing turnover intentions among nurses with two children.

Variables	Regression coefficient	Standard deviation	Standardized coefficient	*t*	*P*
(Constants)	13.407	1.062		12.619	<0.001
Age	−0.379	0.117	−0.08	−3.247	0.001
Night shifts per month	0.295	0.107	0.066	2.766	0.006
Average monthly income	−0.296	0.146	−0.048	−2.03	0.043
Primary caregiver for the children are grandparents	0.641	0.21	0.069	3.047	0.002
Family-to-work conflict	0.091	0.01	0.258	9.369	<0.001
Work-to-family conflict	0.069	0.01	0.191	6.755	<0.001
POS	−0.156	0.033	−0.112	−4.681	<0.001
DES	−0.119	0.034	−0.095	−3.533	<0.001
ANG	−0.078	0.031	−0.068	−2.526	0.012

*R* = 0.547, *R*^2^ = 0.299; after adjustment: *R*^2^ = 0.295, *F* = 64.579, *P* < 0.001.

## 4 Discussion

### 4.1 High level of turnover intention among female nurses with two children

In this study, female nurses with two children had a relatively high turnover intention, with an average score of 2.18 ± 0.66. This level of turnover intention was higher than the findings of general nurses in Chinathan (39). The reason for this disparity may be the added family and childcare responsibilities shouldered by female nurses with two children, which contribute to a greater possibility of considering leaving their current positions.

When examining the various dimensions of turnover intention, turnover intention II received the highest score (2.33 ± 0.71), this is consistent with an Italian study finding by Sasso et al. (40). This dimension relates to the motivation to seek alternative employment, implying that female nurses with two children have a greater inclination to explore other job opportunities. This could be attributed to the multiple roles they fulfill within their families, such as being a wife, daughter, daughter-in-law, and mother. The caring for aging parents and raising children require a significant investment of time and energy. Additionally, the demanding nature of nursing work, combined with frequent night shifts, poses challenges in maintaining a work-life balance, thereby strengthening their desire to pursue alternative job options. These findings underscore the importance for managers to effectively allocate human resources and enhance the personal sense of achievement among female nurses with two children, as a means to reduce turnover intention.

### 4.2 Factors influencing the turnover intention among female nurses with two children

#### 4.2.1 The higher the work-family behavioral role conflict, the higher the turnover intention

In this study, it was observed that the turnover intention among female nurses with two children increased with higher levels of work-family behavioral role conflict. The dimensions of work-to-family conflict and family-to-work conflict were positively correlated with the overall turnover intention score, with coefficients of 0.412 and 0.440, respectively, this is similar to the findings of the other studies (37, 41). These findings suggest that work-family behavioral role conflict can affect the occurrence of turnover behavior among this group of nurses. The family acts as a solid foundation for an individual’s career. However, the inability to strike a balance between family roles and the sacred role of being a nursing professional is a crucial factor driving these nurses to consider quitting their jobs. There is a study (38) also highlighted that healthcare professionals, owing to their heavy workloads, experience higher levels of work–family conflict, leading to increased levels of occupational burnout and, consequently, a higher turnover intention.

Work represents a vital source of income and personal fulfillment, while raising two children disperses their energy and focus, making it challenging for nurses to fully invest in demanding nursing tasks. Consequently, their decreased concentration and inability to meet work expectations may result in dissatisfaction from supervisors and biases from colleagues, which further amplify their intention to seek alternative employment. With social development, the prevalence of information technology and intensified market competition, the boundaries between work and family life have become increasingly blurred and the intrusion of work demands into the family domain has become more prominent (42). A meta-analysis examining the impact of work–family conflict on employees’ work domain showed that work-to-family conflict increases employees’ turnover intention (43). When confronted with work–family conflicts, employees weigh the costs of resolving these conflicts, and changing the work environment is often perceived as easier than altering the family environment.

Consequently, employees may opt to leave their jobs to mitigate the conflicts between work and family responsibilities. To address these challenges, hospital managers should consider the unique circumstances of each department and implement strategies such as flexible scheduling and dynamic allocation of human resources. These measures can alleviate work–family conflict among female nurses with two children, enhance support from their families regarding nursing work, minimize turnover, and retain talented nurses. By fostering an environment that supports work-life balance, healthcare organizations can retain exceptional nursing professionals and provide healthcare services to a wide range of patients.

#### 4.2.2 The higher the levels of regulatory emotional self-efficacy, the lower the turnover intention

In this study, it was discovered that female nurses with two children who had higher levels of regulatory emotional self-efficacy exhibited lower turnover intention. This finding is also supported by a study on registered nurses in New Zealand (44), which confirmed that higher levels of self-efficacy are associated with a reduced turnover intention. Female nurses with high regulatory emotional self-efficacy are likely to have the confidence and ability to effectively cope with the pressures arising from their work and family responsibilities, thereby reducing their inclination to quit due to stress-related factors. Furthermore, female nurses who exhibit greater regulatory emotional self-efficacy are proficient in adjusting their mindset and effectively managing conflicts and pressures in their work environment. This proficiency enables them to navigate the challenges of their profession more effectively and leads to a more positive attitude toward their work and a reduced turnover intention.

The impact of regulatory emotional self-efficacy on turnover intention can be ranked in the following order of significance: POS score, DES score, and ANG score. Importantly, all dimensions of regulatory emotional self-efficacy exert a negative influence, suggesting that higher scores on these dimensions are associated with a lower turnover intention. These findings are consistent with the existing study results (45, 46). It is plausible that female nurses with two children while juggling their family responsibilities and work commitments, may experience moments of sadness, discouragement, anxiety, anger, and frustration. Firstly, being proficient in expressing and utilizing positive emotions can help individuals distance themselves from negative emotions such as anxiety and depression (47). Secondly, based on the theory of self-determination, when individuals find themselves in a prolonged state of being unable to regulate and manage negative emotions, their beliefs and decision-making tend to be negative, thus fostering negative choices like the turnover intention (48). There are also Chinese studies (29) pointed out highlighted the significance of focusing on the psychological wellbeing of nurses in training and cultivating their regulatory emotional self-efficacy to reduce their turnover intention. Furthermore, a study was also noted (49) that employing appropriate emotional therapy can enhance nurses’ ability to manage their emotions effectively and enable them to approach challenges with optimism and seek appropriate ways to express their emotions. As a result, this can ameliorate professional burnout among nurses and reduce turnover. These insights inspire nursing managers to conduct timely mental health education and informative sessions to assist nurses in better managing their emotions, foster an optimistic work environment, and elevate their job satisfaction.

#### 4.2.3 The older the age, the lower the turnover intention

This study revealed an inverse relationship between age and turnover intention among female nurses with two children. Specifically, these nurses aged 35 and below exhibited higher turnover intention scores compared to their older counterparts. Moreover, nurses aged 36 to 40 had higher turnover intention scores than those aged 41 and above. These results are consistent with the results of previous studies (50). Similar trends have also been observed in a study conducted in Turkey, where older nurses were more inclined to remain in the hospital, while younger nurses demonstrated a higher propensity to resign (51).

Remarkably, 81.3% of the survey participants were concentrated in the 31 to 40 age range, which aligns with the demographic composition of the 1980s and 1990s generations. As the mainstream workforce in China, younger individuals within this age range often possess a wealth of innovative ideas but may have lower resilience to stress. Consequently, they are more susceptible to contemplating resignation due to the demanding nature of nursing work and familial pressures (52). Conversely, older nurses, leveraging their extensive work experience and robust professional networks, tend to experience higher job satisfaction. In addition, nurses in this age group, burdened with the financial responsibilities of raising two children, rely heavily on their work income as the primary source of financial stability for their families. Moreover, due to factors such as age, they may encounter challenges in securing alternative employment, which further reduces their turnover intention.

#### 4.2.4 Primary caregivers for children in families

This study found that female nurses with two children whose primary carers were the children’s grandparents had a higher intention to leave than those whose primary carers were themselves and their spouses and those whose careers were the children’s grandparents, which is different from the results of previous studies (53). Previous study (53) have shown that intergenerational care by elders can increase female employment. The possible reason for the findings of this study is that grandparents have become the mainstream in caring for their grandchildren in the Sichuan area. The role of being a daughter who believes that her parents (the child’s grandparents) helping to look after the child is adding to the parents’ problems. Guilt over time forced the nurse to develop the intention of offering to leave her job to care for the child herself.

As a response to this, some hospitals in China have taken proactive steps by organizing summer and winter vacation daycare programs for employees’ children to address childcare needs during specific periods, which showcases a high level of care and consideration for employees. Such measures foster a sense of belonging and job security among nurses, lead to decreased turnover intention, and serve as valuable examples that medical institutions can learn from and implement.

#### 4.2.5 Monthly night shifts

This study showed that female nurses with two children who do not have night shifts exhibit significantly lower scores in their turnover intention, compared to those who have night shifts. Moreover, among female nurses with monthly night shifts, those who work 1–10 shifts per month have lower turnover intention scores than those who work more than 10 shifts per month. Hence, it is evident that there is a correlation between the number of monthly night shifts and the turnover intention of female nurses with two children, which is in line with the research findings from other national studies (54).

The possible reason for this correlation is that frequent night shifts disrupt nurses’ sleep patterns and lead to irregularities in their physical and mental wellbeing, the limited time available for childcare and family responsibilities gives rise to a higher turnover intention. Additionally, a Korean study (55) demonstrated that nurses who regularly work night shifts on rotation often experience various health issues, which subsequently leads to an increased turnover intention. Similarly, research from the United States (56) indicated that night shifts increase risks such as inadequate sleep, heightened family pressures, and emotional fluctuations. These findings enlighten nursing managers to prioritize the wellbeing of nurses working night shifts and provide them with adequate care and support.

#### 4.2.6 Average monthly income

In this study, it was found that female nurses with two children who had an average monthly income of less than 3,500 yuan exhibited a higher turnover intention compared to those with an average monthly income of 3,500 yuan or higher. Additionally, among female nurses with two children, those with an average monthly income ranging from 3,501 to 5,500 yuan had higher turnover intention scores than those with a monthly income of 5,500 yuan or above. These findings align with multiple previous studies (57, 58). For example, a survey conducted by Wu Yino from the Chinese Academy of Medical Sciences (2) evaluating the current status of turnover intention among medical staff in 144 tertiary public hospitals also pointed out the low salary as one of the important contributing factors to nurses’ turnover intention.

Salary and treatment play a crucial role in motivating employees and serve as the fundamental basis for fostering employee engagement and talent retention. Therefore, nursing managers should consider improving nurses’ welfare benefits and compensation levels to increase job satisfaction and reduce turnover intention.

## 5 Limitations

It is crucial to recognize the limitations of this study, which primarily examined the impact of individual internal factors such as general information, work-family behavioral role conflict, and regulatory emotional self-efficacy on turnover intention. The multiple linear regression analysis, which explained only 29.5% of the overall variability in turnover intention scores, indicated the existence of other factors that impact the turnover intention of female nurses with two children. This study is a cross-sectional survey with a single research method. In future investigations, incorporating interview methods and adopting a mixed-methods approach of both qualitative and quantitative measures can further enhance the depth of research in this field. Moreover, additional variables are expected to be included to broaden the scope of investigation.

## 6 Conclusion

The findings of this study revealed a relatively high level of turnover intention among female nurses with two children. There is a positive association between work-family behavioral role conflict and turnover intention, while regulatory emotional self-efficacy demonstrates a negative correlation with turnover intention. As public hospitals steadily progress toward high-quality development, hospital management departments can take initiatives such as organizing psychological workshops and conducting training sessions on emotional regulation and management. These interventions can enhance nurses’ emotional management abilities and enable them to confidently navigate the conflicts between work and family. Consequently, this will help reduce their turnover intention and foster a stable and sustainable nursing workforce.

## Data availability statement

The original contributions presented in the study are included in the article/supplementary material, further inquiries can be directed to the corresponding author.

## Ethics statement

The studies involving humans were approved by the Medical Ethics Committee of the Second People’s Hospital of Yibin, Sichuan, China. The studies were conducted in accordance with the local legislation and institutional requirements. The participants provided their written informed consent to participate in this study.

## Author contributions

CL: Conceptualization, Investigation, Methodology, Writing – original draft, Writing – review & editing. LT: Investigation, Methodology, Writing – review & editing. XW: Writing – review & editing, Supervision, Validation. YW: Writing – review & editing, Data curation. YC: Writing – review & editing, Data curation. LZ: Data curation, Methodology, Supervision, Writing – review & editing.
